# Capnometric feedback training decreases 24-h blood pressure in hypertensive postmenopausal women

**DOI:** 10.1186/s12872-021-02240-x

**Published:** 2021-09-17

**Authors:** David E. Anderson, Alexis N. Reeves, Wolf E. Mehling, Margaret A. Chesney

**Affiliations:** 1 Department of Medicine, School of Medicine, University of California, San Francisco, San Francisco, CA 415-613-7343 USA; 2 Department of Family and Community Medicine, School of Medicine, University of California, San Francisco, San Francisco, USA

**Keywords:** Blood pressure, Breathing, Carbon dioxide, Hypercapnia, Hypertension

## Abstract

**Background:**

High normal resting pCO_2_ is a risk factor for salt sensitivity of blood pressure (BP) in normotensive humans and has been associated with higher resting systolic BP in postmenopausal women. To date, however, no known studies have investigated the effects of regular practice of voluntary mild hypocapnic breathing on BP in hypertensive patients. The objective of the present research was to test the hypothesis that capnometric feedback training can decrease both resting pCO_2_ and 24-h BP in a series of mildly hypertensive postmenopausal women.

**Methods:**

A small portable end tidal CO_2_ (etCO_2_) monitor was constructed and equipped with software that determined the difference between the momentary etCO_2_ and a pre-programmed criterion range. The monitor enabled auditory feedback for variations in CO_2_ outside the criterion range. 16 mildly hypertensive postmenopausal women were individually trained to sustain small decreases in etCO_2_ during six weekly sessions in the clinic and daily sessions at home. 24-h BP monitoring was conducted before and after the intervention, and in 16 prehypertensive postmenopausal women in a control group who did not engage in the capnometric training.

**Results:**

Following the intervention, all 16 capnometric training participants showed decreases in resting etCO_2_ (− 4.3 ± 0.4 mmHg; *p* < .01) while 15 showed decreases in 24-h systolic BP (− 7.6 ± 2.0 mmHg; *p* < .01). No significant changes in either measure was observed in the control group. In addition, nighttime (− 9.5 ± 2.6; *p* < .01) and daytime (− 6.7 ± 0.2 mmHg) systolic BP were both decreased following capnometric training, while no significant changes in nighttime (− 2.8 ± 2.2 mmHg; *p* = .11) or daytime (− 0.7 ± 1.0 mmHg; *p* ≤ .247) systolic BP were observed in the control group.

**Conclusions:**

These findings support the hypothesis that regular practice of mild hypocapnic breathing that decreases resting etCO_2_ reliably decreases 24-h blood pressure in hypertensive postmenopausal women. The extent to which these effects persist beyond the training period or can be observed in other hypertensive subgroups remains to be investigated.

## Introduction

Primary hypertension is chronically elevated blood pressure (BP) of unknown origin. It is the most common cardiovascular disorder, and a major risk factor for stroke and coronary heart disease [1]. Epidemiological research has shown hypertension to be a “disease of civilization” [2], a conclusion supported by studies showing that the rate of rise in BP with age and the incidence of hypertension is higher in women living in urban settings than in cloistered environments, matched for demographic variables and diet [3]. The physiological mechanism by which chronic social stress can increase the set point for BP over time has long eluded clarification, however.

Ever since the discovery of the “fight or flight” response almost a century ago [4], investigators have been interested in the role of the sympathetic nervous system (SNS) in the pathogenesis of primary hypertension. That the maintenance of hypertension involves increased SNS activity in some patients [5, 6] is consistent with this view. Subsequently, clinically oriented studies addressed the blood pressure (BP) effects of a slowed breathing rate that decreases SNS activity. Preliminary successes were reported in several studies which showed that breathing interventions could decrease resting BP (reviewed by Shi et al. [7] and Park et al. [8]). However, none of these studies reported decreases in 24-h BP, leading some to conclude that the era of breathing interventions in hypertension was over [9].

Others had observed, however, that the decreases in rate during breathing interventions are typically offset by proportional increases in tidal volume (breathing depth) that maintain pCO_2_ at or near pre-intervention baselines [10]. Breathing exercises that maintain stable pCO_2_ are not the only possible means by which the set point for BP might be modified. It has been known for many years that voluntary hypocapnic breathing that decreases pCO_2_ also decreases BP acutely [11]. Conversely, it has also been reported that voluntary increases in pCO_2_ within the normal range sustained for 30 min by capnometric feedback increased BP acutely in normotensive men [12]. In neither study was heart rate changed. In addition, previous research has found that high resting pCO_2_ is a risk factor for BP sensitivity to high sodium intake in older [13] and younger [14] normotensive individuals.

To date, no studies have examined the effects of breathing exercises that reliably decrease pCO_2_ on 24-h BP. In the present study, the effects of regular practice of a mild hypocapnic breathing task on BP were investigated in a series of postmenopausal women with hypertensive BP levels. (In this report, the terms hypocapnic (decreased) and hypercapnic (increased) refer to the direction of change in pCO_2_, not levels beyond the normal range.) The selection of women as participants was based on the results of previous research that found a significant positive correlation between resting pCO_2_ and resting BP in postmenopausal women, but not age-matched men [15]. The correlation was found to be greater in women who tended to inhibit the expression of anger [16]. The present study tested the hypothesis that regular practice of a mild hypocapnic breathing exercise by hypertensive postmenopausal women would over time result in decreases in resting etCO_2_ together with decreases in 24-h systolic BP.

## Methods

### Recruitment, screening, and pre-intervention monitoring in the clinic and natural environment

Participants were recruited via the Kaiser Permanente, Northern California (KPNC) health system using a computer algorithm that identified potential candidates via inclusion and exclusion criteria. The inclusion criteria included (a) English speaking females 50–75 years at the time of entry, at least 1 year without menstrual cycle, body mass index (BMI): 19–31, and 24-h systolic BP 140 > 150 mmHg. Exclusion criteria included a history of respiratory, cardiovascular, liver or kidney disease, or diabetes; current medication that influences respiratory function, including benzodiazepines, narcotics or tranquilizers; steroid use within the past 6 months, including hormone replacement therapy; and recent tobacco use. The study was approved by the Institutional Review Boards of University of California, San Francisco (UCSF) and the Kaiser Foundation Research Institute (KFRI). Letters describing the study were mailed to potential candidates. Those who indicated interest were screened by telephone interview to confirm inclusion and exclusion criteria.

Twenty-six candidates were invited for in-person physiological screening. During a first pre-intervention screening session, study objectives and procedures were described, and informed consent was obtained. Monitoring of breath-to-breath respiratory variables occurred during 25 min of seated rest in a recliner chair in a quiet room. etCO_2_ and breathing rate were measured via a nasal cannula attached to a battery-powered respiratory gas monitor that had been customized for continuous recording. To avoid the influence of digestion on pCO_2_, candidates were asked to fast for 2 h before the session. The capnometer was calibrated before each clinic session using a known CO_2_ concentration. Measurements of systolic and diastolic BP and heart rate were recorded every 6 min, using an ambulatory monitor (Space Labs, Model 90207, Redmond, WA). The first BP measurement was discarded and means of the remaining measurements were averaged. After this 25 min session, the candidate wore the ambulatory BP monitor for the next 24 h in the natural environment, during which BP and heart rate were recorded once per hour.

Each candidate returned to the clinic setting within 72 h. If the 24-h SBP or DBP mean was outside the inclusion range, the candidate was excused and referred to her physician, as appropriate. For qualifying candidates, a second 25 min clinic baseline session was conducted, with means of each resting measure calculated as in the first session. Of the 26 participating women, the first 10 were studied with a contingency that involved auditory feedback only when etCO_2_ decreased below the lower limit of the criterion range (three to seven mmHg below the individual resting baseline level). When this contingency proved inadequate to generate consistently effective decreases in etCO_2_ within that range, a more complex contingency was instated whereby auditory feedback was presented whenever etCO_2_ drifted above or below the criterion range. The auditory stimulus was terminated as soon as etCO_2_ returned within the criterion range. The data analyzed in the present study were obtained from the 16 women who were trained under this latter contingency.

Prior to the onset of capnometric feedback training, each participant was advised to maintain their normal daily exercise regimens and diet throughout the study.

### Control group

Data from 16 prehypertensive postmenopausal women with 24 h mean systolic BP between 130 and 140 mmHg were selected from a larger group of women who were serving as control subjects in a different ongoing study. These participants had also been recruited from KPNC and were screened to establish eligibility for participation via review of medical records and a personal interview, as had been conducted for individuals in the capnometric training group. The same inclusion and exclusion criteria were employed. Their baseline and follow-up respiratory and cardiovascular measures were determined by the same methods as for the capnometric feedback group. The interval between these measurements was equivalent to those employed for the capnometric group. These participants were also advised to maintain their normal physical exercise and dietary habits.

### Capnometric feedback training

Participants who qualified for the study completed six weekly clinic training sessions during which they attempted to sustain etCO_2_ five mmHg below individual resting mean levels. Each training session was preceded by a 10 min rest interval, followed by a 25-min period during which the feedback contingency was in effect. At the detection of each new breath, the capnometer calculated mean etCO_2_ for all breaths during the preceding 15 s. Each recorded value was compared with the criterion range that was 3–7 mmHg below the individual baseline mean etCO_2_. When the observed 15 s mean etCO_2_ drifted above or below the criterion range, a non-aversive auditory stimulus was onset that remained in effect until etCO_2_ re-entered the criterion range.

During the first training session, an instructor remained in the room with the participant, encouraging her to adjust breathing depth as needed to stay within the criterion range of etCO_2_ while maintaining a normal breathing rate. During all subsequent training sessions, the participant engaged in the feedback training task while alone. During each session, etCO_2_ and breathing rate were determined from inter-breath intervals, while systolic and diastolic BP, and heart rate, were recorded every 6 min.

Following each clinic session, the participant took the portable etCO_2_ monitor home for regular practice sessions, 5 days per week. As during clinic sessions, the participant was instructed to rest for 10 min preceding the 25 min training sessions at home. During the home sessions, etCO_2_ and breathing rate were monitored continuously, but BP and heart rate were not recorded.

### Post-intervention monitoring in the natural environment

After completion of the capnometric training sessions, a post-intervention 25 min rest session was completed in the clinic, during which etCO_2_ and breathing rate were recorded, while systolic and diastolic BP and heart rate were monitored every 6 min. Following this session, ambulatory BP and heart rate were monitored once per hour for the next 24 h in the natural environment. During this 24-h post-intervention period, etCO_2_ and breathing rate were recorded during a 25 min rest session at home. The means of the clinic and home rest sessions were averaged, providing post-intervention outcome data.

### Data analysis

The analysis of the data included calculation of the significance of the correlation coefficients for successive means for each measure across sessions for the capnometric group with training session number. The significance of the differences between baseline and post-intervention means for each measure was determined via two-tailed t-tests for independent samples for both the capnometric training and control group. The significance of the differences between groups in each measure was determined via two tailed t-tests (Tables 2 and 3) and chi square for consistency of direction of change (Table 2). STATA Version 12 was used to perform the statistical tests.

## Results

### Baseline demographics and physiological measures for the intervention and control groups

Table 1 shows that baseline means of age, body mass index, body surface area, resting etCO_2_, resting breathing rate, 24-h diastolic pressure and 24-h heart rate for the capnometric training group and the control group were not significantly different. Baseline mean 24-mmHg systolic pressure was higher in the capnometric feedback group than in the control group. However, baseline daytime mean systolic BP in the capnometric feedback group was not significantly different from that in the control group (see Table 3).

**Table 1:** Means and standard errors of demographic and physiological measures at baseline for the capnometric training intervention (n = 16) and the control group (n = 16)

Measure	Capnography training group (n = 16)	Control group (n = 16)	*Δp* value
Age	69.0 ± 1.5	64.0 ± 2.0	.122
Body mass index (kg/m^2^)	24.9 ± 0.9	26.3 ± 0.7	.236
Body surface area (BSA)	1.7 ± 0.1	1.7 ± 0.0	.703
End tidal CO_2_ (mmHg)	37.0 ± 0.9	37.0 ± 0.6	.740
Breathing rate (/min)	14.8 ± 0.6	14.4 ± 0.8	.643
Systolic BP (mmHg)	144.2 ± 0.9	138.3 ± 0.4	.001*
Diastolic BP (mmHg)	78.4 ± 1.9	78.2 ± 1.4	.486
Heart rate (bpm)	67.0 ± 1.3	70.6 ± 1.9	.166

* = *p* < .01

### Performance

**Fig. 1:**
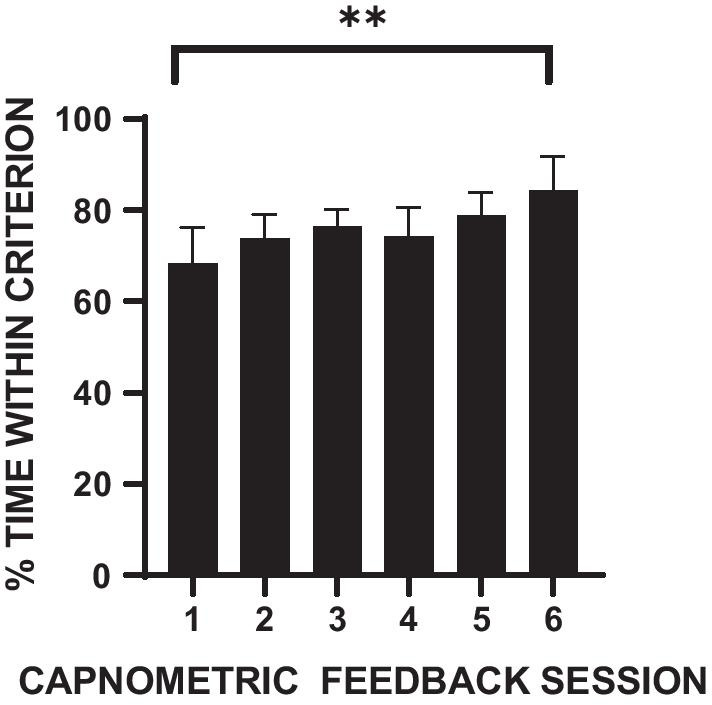
Means and standard errors of the percentage time within the end tidal CO_2_ criterion range across six weekly capnometric feedback training sessions for 16 participants in the capnometric training group. **p < .01

Figure 1 shows that percent time breathing within the criterion range for the feedback group increased progressively from the first to the sixth clinic training session (r = 0.92; *p* < 0.01).

### EtCO_2_ and breathing rate in the capnometric training group

**Fig. 2:**
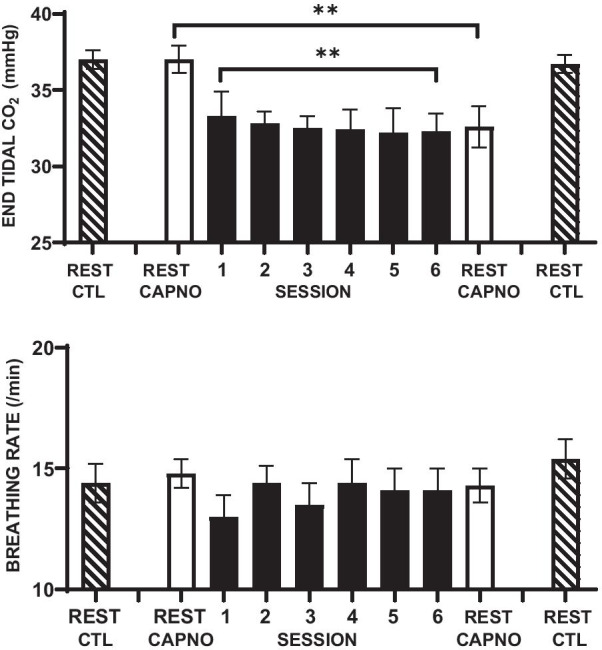
Means and standard errors of end tidal CO_2_ (upper panel) and breathing rate (lower panel) during baseline and post-intervention rest (white bars), six weekly clinic sessions of capnometric training (black bars), and preceding and following the control period (hatched bars). **p < .01

The upper panel of Fig. 2 shows a significant downward trend in mean etCO_2_ over the course of the six clinic feedback sessions (r =  − 0.90; *p* .01). It also shows significant differences in resting etCO_2_ between baseline and post-intervention monitoring in the feedback group, but not in the control group (Fig. 2 and Table 2). The decrease in etCO_2_ in the feedback group was significantly greater than in the control group (Table 2).

**Table 2:** Means and standard errors (S.E.) of each physiological measure before and after the capnometric training intervention (n = 16) and in the control group (n = 16), the significance of the differences in the post-intervention changes in each measure, and the significance of the differences between capnometric training and control groups

Group	Baseline mean ± S.E	After mean ± S.E	∆ (Mean ± S.E	*p*	Between group **∆**	
					X^2^	*p*
*Capnometric Group*						
End tidal CO_2_	37.0 ± 0.9	32.6 ± 1.4	− 4.3 ± 0.4	*p* < .01	11.22	*p* < .01
Breathing rate	14.8 ± 0.6	14.3 ± 0.7	− 0.5 ± 0.2	*p* < .01	0.13	*p* = .72
Systolic BP	144.2 ± 0.9	136.6 ± 1.1	− 7.6 ± 2.0	*p* < .01	6.78	*p* < .01
Diastolic BP	78.4 ± 1.9	76.8 ± 1.7	− 1.6 ± 1.3	*p* = .42	0.58	*p* = .44
Heart rate	67.0 ± 1.7	67.9 ± 0.1	+ 3.7 ± 1.8	*p* = .62	0.14	*p* = .71
*Control group*						
End tidal CO_2_	37.0 ± 0.6	36.7 ± 0.6	− 0.3 ± 0.1	*p* = .43		
Breathing rate	14.4 ± .08	15.4 ± 0.8	+ 1.0 ± 0.9	*p* = .14		
Systolic BP	138.3 ± 0.4	137.0 ± 1.3	− 1.3 ± 1.3	*p* = .65		
Diastolic BP	78.2 ± 1.4	78.2 ± 1.3	0.0 ± 1.2	*p* = .65		
Heart rate	70.6 ± 1.9	71.5 ± 2.0	+ 0.9 ± 1.1	*p* = .52		

End tidal CO_2_ = mmHg, Breathing rate = breaths/minute, Systolic and Diastolic BP = mmHg, Heart rate = bpm, ∆ = difference score, X^2^ = Chi square

The lower panel of Fig. 2 shows no significant trend in mean breathing rate over the course of the six clinic feedback sessions (r = 0.53; *p* = 0.36). However, breathing rate following the intervention was decreased from baseline values (Table 2). Figure 2 and Table 2 show no significant differences between groups in breathing rate between baseline and follow-up monitoring (Table 2).

### Systolic and diastolic blood pressure and heart rate in the capnometric training group

**Fig. 3:**
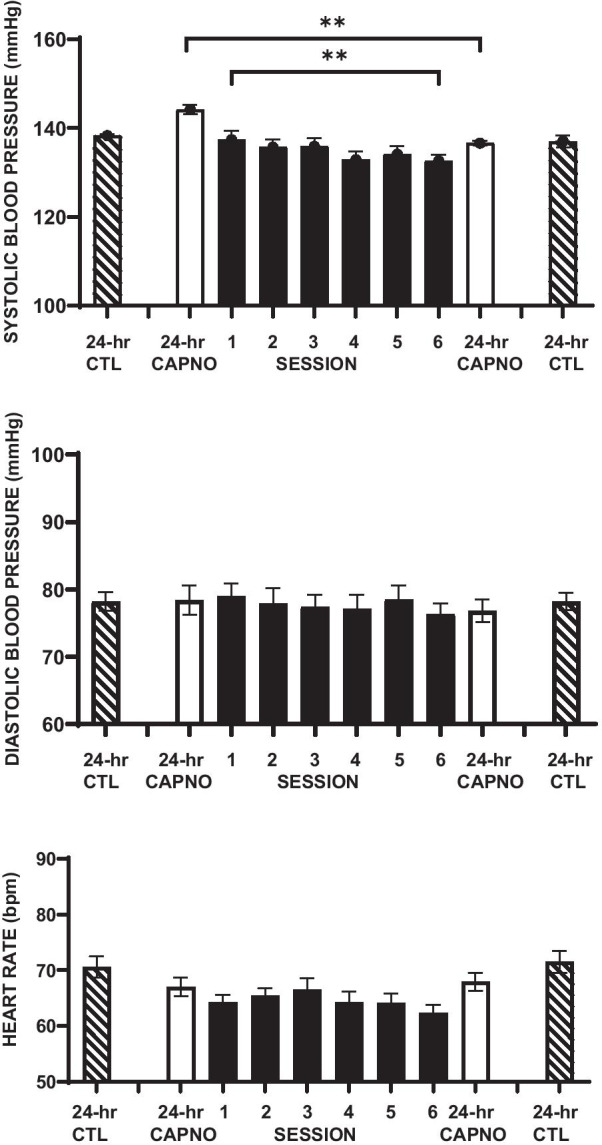
Means and standard errors of 24 h systolic blood pressure (upper panel), diastolic blood pressure (middle panel), and heart rate (lower panel) preceding and following the capnometric training intervention (white bars), during the six training sessions (black bars) and preceding and following the control period (hatched bars). **p < .01, *p < .05

The upper panel of Fig. 3 shows that group mean systolic BP decreased progressively across the six clinic training sessions (r = 0.91; *p* < .01). Table 2 also shows that 24-h mean systolic BP was lower during post-intervention monitoring than during baseline monitoring (Table 2). It also shows that the probability of a decrease in systolic BP in the intervention group was significantly greater than in the control group (X^2^ = 5.7; *p* < .009).

The middle panel of Fig. 3 shows no significant trend in diastolic BP change across the six clinic training sessions (r =  − 0.73; *p* > 0.145), and that group 24-h diastolic mean BP was not significantly different during post-intervention monitoring than at baseline (Table 2).

The lower panel of Fig. 3 shows no significant trend in mean heart rate change across the six clinic training sessions (r =  − 0.53; *p* > 0.229), no significant change in breathing rate following the intervention, and no significant difference between groups (Table 2).

### Respiratory and cardiovascular changes in the control group and between groups

Figure 2 and Table 2 show no significant mean changes in the control group in resting etCO_2_ or breathing rate during follow-up clinic monitoring. They also show no significant changes in 24-h systolic or diastolic pressure, or 24-h heart rate in the control group. The differences between capnometric training and control groups in mean changes in etCO_2_ and 24-h systolic BP were both significant (Table 2). The differences between groups in the changes in clinic breathing rate, 24-h diastolic BP, and heart rate were not significant.

### Blood pressure and heart rate changes during the nighttime and daytime

The upper section of Table 3 shows means and standard errors of systolic and diastolic pressure, and heart rate, for the 8-h night and 16 h day in the capnometric feedback group following the intervention. Significant pre-post changes in systolic pressure were observed during the nighttime and daytime, and in diastolic pressure during the nighttime. Group differences in change in nighttime and daytime systolic pressure were significant, as were the changes for nighttime diastolic pressure. No significant nighttime or daytime changes in heart rate were observed.

**Table 3:** Means and standard errors for systolic and diastolic blood pressure, and heart rate during nighttime and daytime ambulatory monitoring of 16 hypertensive and 16 control post-menopausal women

	Baseline	After	**∆**	*p*	Between group **∆**
*Capnometric training*					
Systolic BP					
Night	137.0 ± 3.3	127.5 ± 2.6	− 9.5 ± 2.6	*p* < .001	*p* < .03
Day	147.8 ± 3.0	141.1 ± 3.2	− 6.7 ± 0.2	*p* < .001	*p* < .01
Dipping	10.8 ± 2.8	13.6 ± 2.2	− 2.8 ± 3.1	*p* < *.*191	*p* < .19
Diastolic BP					
Night	75.1 ± 2.1	70.6 ± 2.0	− 4.5 ± 1.1	*p* < .001	*p* < .03
Day	82.8 ± 1.8	80.0 ± 1.9	− 2.3 ± 1.5	*p* < .784	*p* < .19
Dipping	7.1 ± 2.0	9.4 ± 1.3	− 2.3 ± 1.5	*p* < .073	*p* < .01
Heart rate					
Night	63.3 ± 1.1	65.2 ± 0.9	1.8 ± 1.2	*p* < .077	*p* < .10
Day	68.9 ± 1.8	69.3 ± 1.4	0.4 ± 1.2	*p* < .372	*p* < .72
Dipping	5.5 ± 1.0	4.1 ± 1.1	− 1.4 ± 1.2	*p* < .132	*p* < .33
*Control*					
Systolic BP					
Night	129.0 ± 2.8	126.2 ± 2.7	− 2.8 ± 2.2	*p* < .112	
Day	142.9 ± 2.4	142.4 ± 3.3	− 0.7 ± 1.0	*p* < .247	
Dipping	14.5 ± 2.8	17.4 ± 1.8	2.8 ± 2.8	*p* < *.*166	
Diastolic BP					
Night	72.0 ± 2.1	71.0 ± 2.0	− 1.0 ± 1.2	*p* < .209	
Day	81.2 ± 2.1	81.8 ± 2.5	0.6 ± 1.6	*p* < .354	
Dipping	10.0 ± 1.9	7.2 ± 1.8	7.2 ± 2.2	*p* < .002	
Heart rate					
Night	65.9 ± 1.3	66.7 ± 1.2	0.8 ± 1.3	*p* < .275	
Day	72.9 ± 1.3	74.0 ± 1.8	1.0 ± 1.2	*p* < .209	
Dipping	7.3 ± 1.6	6.9 ± 1.2	0.4 ± 1.4	*p* < .391	

Systolic and Diastolic BP = mmHg, Heart rate = bpm

Δ = difference score

The lower section of Table 3 shows means and standard errors of nighttime and daytime systolic and diastolic pressure and heart rate in the control group. No significant pre-post changes in 24-h systolic, diastolic pressure or heart rate were observed during the day or at night for either group.

## Discussion

This study found that 6 weeks of regular practice of hypocapnic breathing reliably decreased 24-h systolic BP in postmenopausal hypertensive women. This is the first application of capnometric feedback applied to hypertensive patients, but far from the first to investigate the effects of instructed deep breathing on BP. Thirteen studies of regular deep breathing effects on resting BP have been reviewed recently [17]. In general, the procedures in those studies involved instructions to breathe deeply at a normal rate. Most reported decreases in resting BP and heart rate and concluded that the effects must have been mediated by decreases in sympathetic nervous system (SNS) activity. Three measured 24-h BP but found no significant BP changes following the intervention. None measured pCO_2_. The present study differs from previous studies in that the sessions were longer in duration (25 min rather than 10 min), recorded etCO_2_ continuously, and employed contingencies for the maintenance of breathing depths within or near the normal range of etCO_2_. Since 24-h ambulatory heart rate was increased following this intervention, the findings are consistent with the conclusion that the changes in 24-h BP were mediated via decreases in peripheral vascular resistance.

24-h blood pressure monitoring is considered a better index of the set point than resting BP, because it is less susceptible to the confounding effects of “white coat hypertension” and “masked hypertension” and is more prognostic of heart and vascular disease [18]. A sub-normal magnitude of nocturnal “dipping” in BP from daytime levels has been shown to be a risk factor for cardiovascular morbidity and mortality, independently of BP level [19, 20]. In the present study, nocturnal “dipping” of BP before and after the capnometric feedback training was compared with that in the control group. Capnometric feedback training decreased the systolic BP during the day and at night in the feedback group, but no significant day or night changes were observed in the control group. No significant changes in “dipping” of nighttime blood pressure were observed.

The association between the changes in pCO_2_ and BP found in this study can also be seen in other disorders. For example, obstructive sleep apnea is associated with recurrent episodes of both hypoxia and hypercapnia [21]. Hypoxia increases SNS activity acutely [22], but repeated hypercapnic episodes can also over time increase daytime resting pCO_2_ [23]. In addition, respiratory failure in patients with chronic obstructive pulmonary disease has been associated with increases in both pCO_2_ and BP, and successful treatment is accompanied by a return of both pCO_2_ and BP to pre-failure levels [24].

Individual differences in resting pCO_2_ are a function of several psychological factors. Lower levels have been associated with anxiety [25, 26], while higher levels have been associated with a tendency to worry [16]. Resting pCO_2_ is known to decrease with age, perhaps in response to age-associated decreases in plasma pH [27]. In addition, it tends to be higher in African Americans than Caucasian Americans [28]. The set point for pCO_2_ can change over time in a process known as allostasis, which can override the corrective action of homeostasis [29, 30]. For example, when resting etCO_2_ was measured 7 days and 6 months after an initial baseline session, no significant group mean changes were observed, but test–retest correlations were lower after 6 months than after 1 week [31]. Previous studies of capnometric feedback training to increase low normal resting etCO_2_ have shown it to be effective in ameliorating panic disorder [32, 33] and asthma [34].

In the natural environment, acute changes in pCO_2_ can occur under two behavioral conditions that might over time contribute to disease pathogenesis (reviewed by Sikter et al. [35, 36]). First, acute *increases* in pCO_2_ occur when increases in parasympathetic activity decrease heart rate and ventilation during sustained attention to the external environment. Sustained vigilance serves as a brake on the motor system and prepares the individual for fight or flight [37]. It can be of brief duration or extended over time by experience and conditioning. Second, acute *decreases* in pCO_2_ occur in a context of SNS arousal which redistributes blood flow to the heart, lungs, and skeletal muscle to support fight or flight. For many years, fight or flight was believed by some to participate in the pathogenesis of hypertension [38], but less attention was paid to the role of the increased pCO2 during sustained vigilance as a possible mediating mechanism.

Regarding design, this study sought to determine the consistency with which behaviorally engineered changes in one physiological variable reliably resulted in changes in another physiological variable in individual patients. That all 16 participants showed decreases in resting etCO_2_ while 15 showed post-intervention decreases in 24-h systolic BP indicates a strong association between the two. The untreated control group of postmenopausal women was added to document that repeated measurements of 24-h BP separated in time by at least 4 weeks do not result in significant changes in 24-h BP in the absence of an intervention [39].

A limitation of the study is that the findings apply only to one subgroup of patients, namely, hypertensive postmenopausal women. They cannot necessarily be extended to men. When estrogen is diminished during menopause, the incidence of hypertension in women rises to become comparable to that of men [40]. In older persons, the association between resting pCO_2_ and resting BP is significant in women, but not in men [15]. Whether capnometric feedback of etCO_2_ might decrease 24-h BP in older hypertensive men needs to be investigated.

Another limitation of the study is that the intervention and control groups had different baseline systolic BP levels, but comparable (i.e., prehypertensive) levels following the intervention or control wait-list period. The development of the portable etCO_2_ monitor and associated methodology required to document the effects of capnometric feedback training did not leave adequate time for recruitment of another group of unmedicated hypertensives fitting the inclusion and exclusion criteria within the grant funded study period. However, a group of unmedicated prehypertensive postmenopausal women with identical inclusion and exclusion criteria was available who had previously participated as controls in a different study. It should be noted that the daytime BP at baseline in the control group in the present study was not significantly different from that in the intervention group (Table 3). Other research on breathing interventions in hypertension have provided comparable findings involving hypertensive control groups that had shown no significant changes in 24-h mean BP following the wait list period [41, 42].

A third limitation of the study is that the findings apply only to the time period during which the scheduled practice of hypocapnic breathing was in effect. Follow up data will be needed to determine whether the decreases in 24-h BP persist beyond the termination of the intervention.

More generally, hypertension is thought to be a disorder of long-term sodium regulation involving an interaction between dietary sodium intake and factors that impair the ability of the kidneys to restore and maintain normal plasma volume and/or vascular tone [43–45]. The salt sensitive form of hypertension has been reported to occur more frequently in women than in men [46]. Previous studies have not examined the role of resting pCO_2_ in the maintenance of BP in hypertensive patients, but a role for pCO_2_ in the pathogenesis of a salt sensitive form of experimental hypertension has been documented in studies with large laboratory animals [47]. Together, these findings suggest that capnometric feedback training that decreases resting pCO_2_ might be effective in the management of salt-sensitive forms of primary hypertension.

## Conclusions

This study found that 6 weeks of daily sessions of capnometric feedback training that decreased resting etCO_2_ in hypertensive postmenopausal women was accompanied by significant decreases in systolic 24-h blood pressure, a marker for long-term BP. The study was made possible by the development of a portable etCO_2_ monitor equipped with software that determined whether current etCO_2_ was within a pre-programed criterion range and provided contingent auditory feedback. This new technology has the potential to provide a non-pharmacological intervention for some groups of hypertensive patients that might be useful in the management of this most common cardiovascular disorder.

## Data Availability

The datasets used and/or analyzed during the current study are available from the corresponding author on reasonable request.

## References

[1] Kearney PM, Whelton M, Reynolds K, Whelton PK, He J (2004). Worldwide prevalence of hypertension: a systematic review. J Hypertens.

[2] Korner P (2007). Essential hypertension and its causes: neural and non-neural mechanisms.

[3] Timio M, Verdecchia P, Venanzi S, Gentili S, Ronconi M, Francucci B (1998). Age and blood pressure changes: a 20-year follow-up study in nuns in a secluded order. Hypertension.

[4] Cannon WB (1932). The wisdom of the body.

[5] Fisher JP, Paton JFR (2012). The sympathetic nervous system and blood pressure in humans: implications for hypertension. J Hum Hypertens.

[6] Grassi G, Ram VS (2016). Evidence for a critical role of the sympathetic nervous system in hypertension. J Am Soc Hypertens.

[7] Shi L, Zhang D, Wang L, Zhuang J, Cook R, Chen L (2017). Meditation and blood pressure: a meta-analysis of randomized clinical trials. J Hypertens.

[8] Park S, Han KS (2017). Blood pressure response to meditation and yoga: a systematic review and meta-analysis. J Altern Complement Med.

[9] van Djik PR, van Hateren KJJ, Kleefstra N, Landman GWD (2018). It is time to close the book on device-guided slow breathing. Blood Press.

[10] Conrad A, Müller S, Doberenz S, Kim S, Meuret AE, Wollburg E (2007). Psychophysiological effects of breathing instructions for stress management. Appl Psychophysiol Biofeedback.

[11] Burnum JF, Hickam JB, McIntosh HD (1954). The effect of hypocapnia on arterial blood pressure. Circulation.

[12] Bagrov AY, Federova OV, Austin-Lane JL, Dmitrieva RI, Anderson DE (1995). Endogenous marinobufaenin-like immunoreactive factor and Na^+^K^+^ATPase inhibition during voluntary hypoventilation. Hypertension.

[13] Anderson DE, Dhokalia A, Parsons DJ, Bagrov AY (1996). High end tidal CO_2_ association with blood pressure response to sodium loading in older adults. J Hypertens.

[14] Anderson DE, Dhokalia A, Parsons DJ, Bagrov AY (1998). Sodium sensitivity in young adults with high resting end tidal CO_2_. J Hypertens.

[15] Anderson DE, Parsons DJ, Scuteri A (1999). End tidal CO_2_ is an independent determinant of systolic blood pressure in women. J Hypertens.

[16] Scuteri A, Parsons DJ, Chesney MA, Anderson DE (2001). Anger inhibition potentiates the association of high end-tidal CO_2_ with blood pressure in women. Psychosom Med.

[17] Yau KK, Loke AY (2021). Effects of diaphragmatic deep breathing exercises on prehypertensive or hypertensive adults: a literature review. Complement Ther Clin Pract.

[18] Schwartz JE, Burg MM, Shimbo D, Broderick JE, Stone AA, Ishikawa J (2016). Clinic blood pressure underestimates ambulatory blood pressure an untreated employer-based US population: results from the masked hypertension study. Circulation.

[19] di Raimondo D, Miceli G, Casucci A, Tuttolomondo A, Butta C, Zappulla V (2016). Does sympathetic overactivation feature all Hypertension? Differences of sympathovagal balance according to night/day blood pressure ratio in patients with essential hypertension. Hypertens Res.

[20] Yano Y, Kario K (2021). Nocturnal blood pressure and cardiovascular disease: a review of recent advances. Hypertens Res.

[21] Peppard PE, Young E, Palta M, Skatrud J (2000). Prospective study of the association between sleep-disordered breathing and hypertension. New Engl J Med.

[22] Parati G, Lombardi C, Narkiewicz K (2007). Sleep apnea: epidemiology, pathophysiology, and relation to cardiovascular risk. Am J Physiol Regul Integr Comp Physiol.

[23] Norman RG, Goldring M, Claim JM, Oppenheimer BW, Charney AN, Rapoport DM (2006). Transition from acute to chronic hypercapnia in patients with periodic breathing: predictions from a computer model. J Appl Physiol.

[24] Fontana F, Bernardi P, Tartuferi L (2000). Mechanisms of hypertension in patients with chronic obstructive pulmonary disease and acute respiratory failure. Am J Med.

[25] Van den Bergh O, Zaman J, Bresseleers J, Verhamme P, Van Diest I (2013). Anxiety, pCO_2_ and cerebral blood flow. Int J Psychophysiol.

[26] Davies CD, Craske MG (2014). Low baseline pCO_2_ predicts poorer outcome from behavioral treatments: evidence from a mixed anxiety disorders sample. Psychiatry Res.

[27] Frassetto L, Sebastian A (1996). Age and systemic acid-base equilibrium: analysis of published data. J Gerontol A Biol Sci Med Sci.

[28] Anderson DE, Scuteri A, Agalakova N, Parsons DJ, Bagrov AY (2001). Racial differences in resting end-tidal CO_2_ and circulating sodium pump inhibitor. Am J Hypertens.

[29] Ramsay DS, Woods SC (2014). Clarifying the roles of homeostasis and allostasis in physiological regulation. Psychol Rev.

[30] James G (2019). The adaptive value and clinical significance of allostatic blood pressure variations. Curr Hypertens Rev.

[31] Dhokalia A, Parsons DJ, Anderson DE (1998). Resting end tidal CO_2_ association with age, gender, personality. Psychosom Med.

[32] Tolin DF, McGrath PB, Hale LR, Weiner DN, Gueorgieva R (2017). A multisite benchmarking trial of capnometry guided respiratory intervention for panic disorder in naturalistic treatment settings. Appl Psychophysiol Biofeedback.

[33] Davies CD, McGrath PB, Hale LR, Weiner DN, Tolin DF (2019). Mediators of change in capnometry guided respiratory intervention for panic disorder. Appl Psychophysiol Biofeedback.

[34] Ritz T, Rosenfield D, Steele AM, Millard MW, Meuret A (2014). Controlling asthma by training of capnometry-assisted hypoventilation (CATCH) vs slow breathing, a randomized controlled trial. Chest.

[35] Sikter A, Rihmer Z, de Guevara R (2017). New aspects in the pathomechanism of diseases of civilization. Part 1. Theoretical background of a hypothesis. Neuropsychopharmacol Hung.

[36] Sikter A, Rihmer Z, de Guevara R (2017). New aspects in the pathomechanism of diseases of civilization. Part 2. Chronic hypocapnia and hypercapnia in medical practice. Neuropsychopharmacol Hung.

[37] Roelofs K (2017). Freeze for action: neurobiological mechanisms in animal and human freezing. Philos Trans R Soc Lond B Biol Sci.

[38] Alexander F (1939). Emotional factors in essential hypertension. Psychosom Med.

[39] Bo Y, Kwok K, Chung V, Yu C-P, Tsoi KK, Wong SY, Lee EK (2020). Short-term reproducibility of ambulatory blood pressure measurements: a systematic review and meta-analysis of 35 observational studies. J Hypertens.

[40] Reckelhoff JF (2001). Gender differences in blood pressure regulation. Hypertension.

[41] de Barros SJL, da Silva GV, de Gusmao TG, de Souza DR, Cardoso CV, Oneda B, Mion D (2017). Effects of long term device-guided slow breathing on sympathetic nervous system activity. Blood Press.

[42] Blom K, Baker B, How M, Dai M, Irvine J, Abbey S (2014). Hypertension analysis of stress reduction using mindfulness meditation and yoga: results from the HARMONY randomized clinical trial. Am J Hypertens.

[43] Weinberger MH (1996). Salt sensitivity of blood pressure in humans. Hypertension.

[44] Cowley AW (1992). Long-term control of arterial blood pressure. Physiol Rev.

[45] Feng W, Dell’Italia LJ, Sanders PW (2017). Novel paradigms of salt and hypertension. J Am Soc Nephrol.

[46] Nestel PJ, Clifton PM, Noakes M, McArthur R, Howe PR (1993). Enhanced blood pressure response to dietary salt in elderly women, especially those with small waist: hip ratio. J Hypertens.

[47] Anderson DE, Chesney MA. Inhibited breathing and salt sensitive hypertension in women. In: Orth-Gomer K, Schneiderman N, Vaccarino V, Deter HC, editors. Psychosocial stress and cardiovascular disease in women. New York: Springer; 2015. p. 181–96.

